# Supplementary material for: Microwave a.c. conductivity of domain walls in ferroelectric thin films
Source: Nat Commun. 2016 May 31;7:11630. doi: 10.1038/ncomms11630 (PMC4895023; doi:10.1038/ncomms11630)
Supplement: Supplementary Information — Supplementary Figures 1-10 [file ncomms11630-s1.pdf]

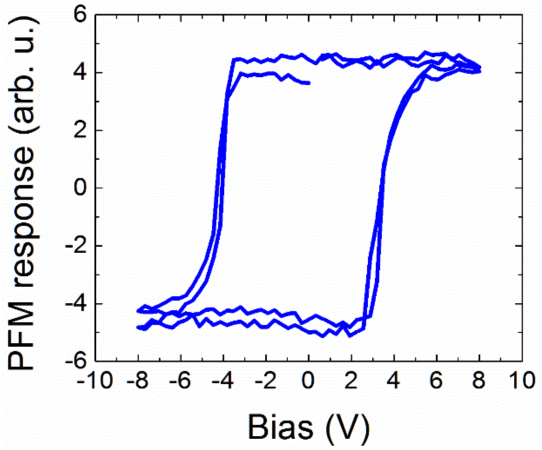

Supplementary Figure 1 | Hysteresis loop of remnant piezoresponse of the pristine PZT film.

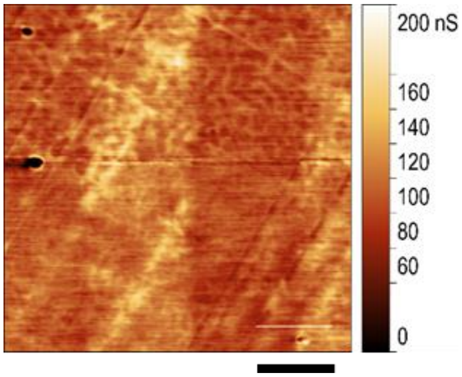

Supplementary Figure 2 | sMIM-*C* image over the stripe domain structure in the pristine PZT film. The image was acquired at a zero dc bias. There is no contrast seen between domain walls and domain bulk. A slight contrast from the stripe domain structure can be caused by a small built-in or residual field in the film. Scale bar is 1 μm.

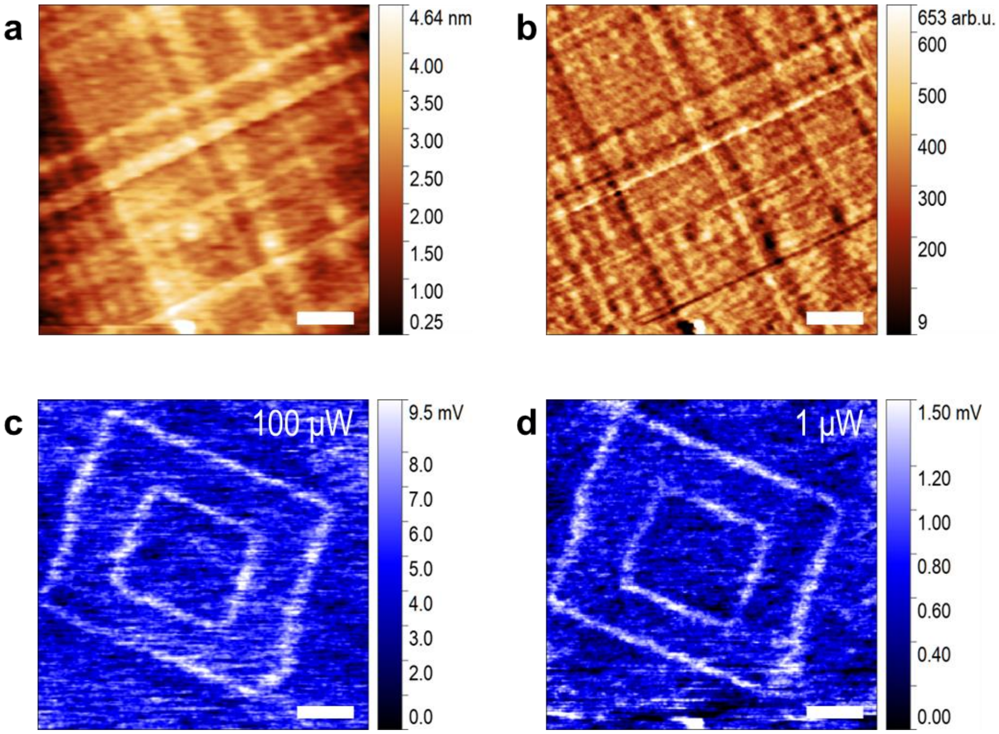

Supplementary Figure 3 | Independence of the domain wall response on the microwave power level. (**a**-**c**) Simultaneously acquired images of surface topography, deflection error, and uncalibrated sMIM-*G* signal, respectively. The microwave power level during acquisition of the image in **c** was to 100 μW (-10 dBm). (**d**) Uncalibrated sMIM-*G* image of the same area acquired immediately after images in **a**-**c** at the microwave power level of 1 μW (-30 dBm), which corresponds to a 10-fold reduction of the ac voltage amplitude at the probe in comparison with the image in **c**. Based on the source power, it can be estimated that the voltage amplitude at the probe tip does not exceed *ca.* 300 mV_{ac} with a 100 μW power and *ca.* 30 mV_{ac} at 1 μW. The uncalibrated sMIM signal in **c** and **d** is approximately proportional to the microwave ac voltage amplitude. As seen the response of the domain walls in respect to the domain bulk is roughly 6 times larger in terms of the output voltage with 100 μW than with 1 μW, which can be concluded from the ranges of the color scales near the images. The image in **d** was de-noised with use of FFT and Gaussian filtering. Scale bars are 1 μm.

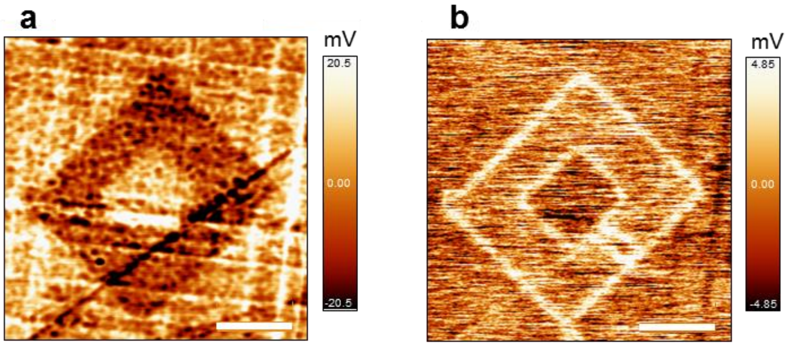

Supplementary Figure 4 | Visualization of domains owing to dielectric tunability. (**a**) Uncalibrated sMIM-*C* image and (**b**) uncalibrated sMIM-*G* image of a box-in-a-box domain structure drawn on the pristine PZT film. The dc bias voltages used to draw the structure were V_{bias} = -7 V and +7 V. A dc bias of +2 V was applied to the tip during imaging. The sMIM-*C* image in **a** shows a contrast from domains due to dielectric tunability of the PZT film and, hence, a difference in dielectric permittivity of up and down domains under the electric field of the probe. Scale bars are 2 μm.

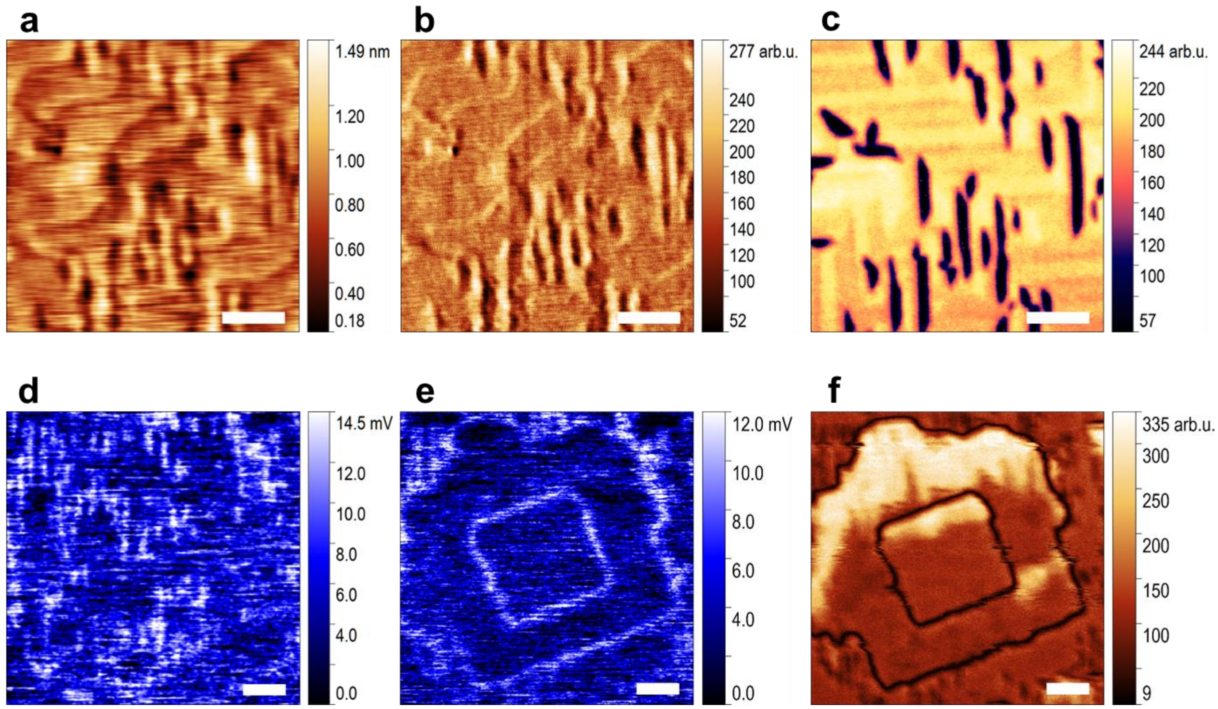

Supplementary Figure 5 | PFM and sMIM images of a $BiFeO_3$ film. (**a-c**) Higher resolution images of surface topography, deflection error, and combined out-of-plane PFM response, respectively, of pristine surface of a $BiFeO_3$ film. The images were acquired simultaneously using a sharper sMIM tip. In the PFM image in **c**, there are clearly seen elongated spontaneous domains (black areas). (**d**) A lower resolution

uncalibrated sMIM-*G* image of the BFO film revealing that the spontaneous domains or domain walls are conducting. The image resolution is insufficient to distinguish between wall and domain conductivity. (**e**) Uncalibrated sMIM-*G* image of a box-in-box domain structure written in the area shown in **d**. (**f**) Out-of-plane PFM amplitude image from the box-in-box domain structure shown in **e**. As seen in **e**, domain walls are conducting. The box-in-box domain structure in **e** and **f** was formed by applying V_{bias} = -6 V and +6 V to the scanning probe. The scale bars are 500 nm in **a-c** and 1 μm in **d-f**.

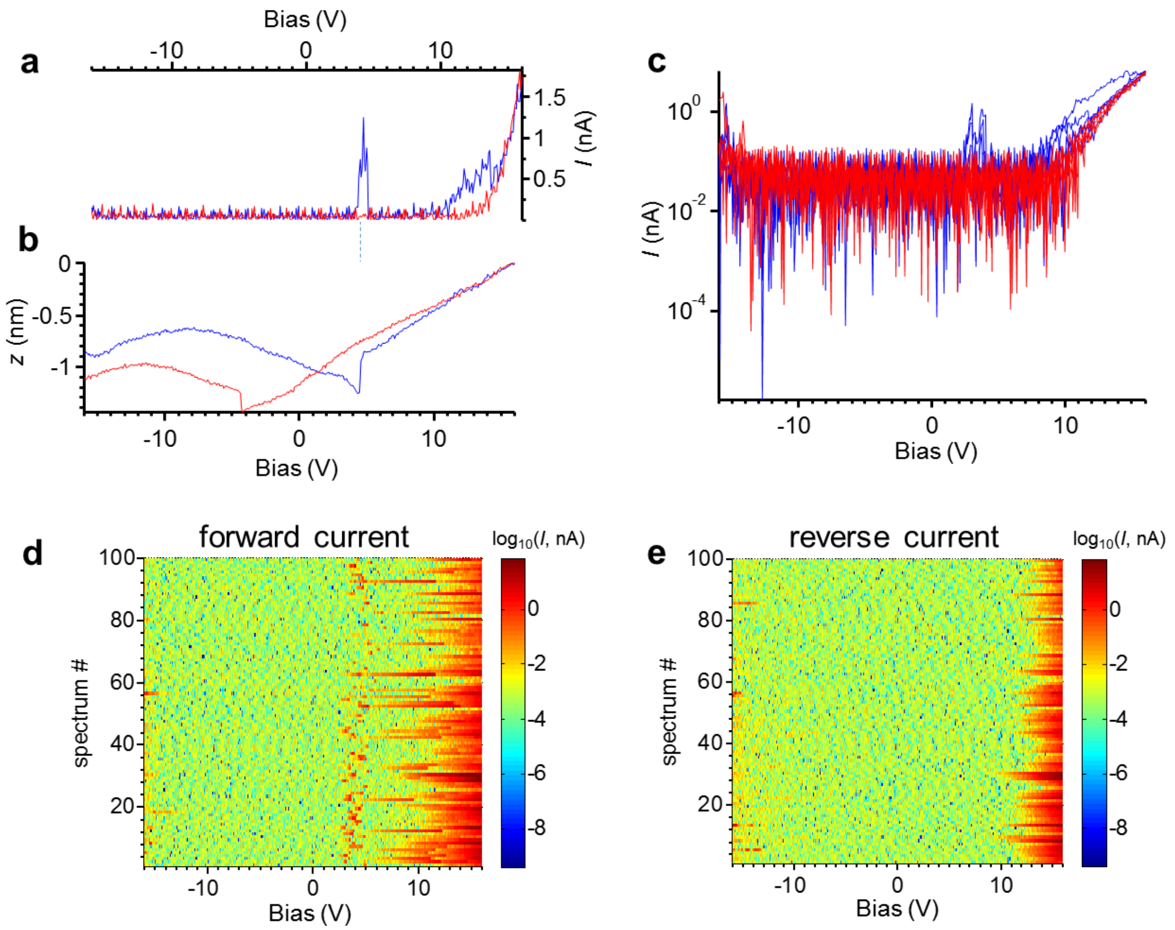

Supplementary Figure 6 | Local dc current-*vs.*-voltage curves of pristine PZT film. (**a**,**b**) Hysteresis loops of dc current trace and piezoelectric strain, respectively, as functions of voltage across the film. Ferroelectric switching is detected as a discontinuous change of piezostrain at *ca*. 5 V and -4.5 V. Although the film is insulating in the almost whole range of probed voltages, at 5 V, there is a clear current peak coinciding with the switching event, which indicates formation of a transient conducting domain wall. (**c**) Representative I-V curves from random locations on the PZT film surface. In **a-c**, the forward and reverse

curves are highlighted by blue and red colors, respectively. (**d**,**e**) 2D maps of forward, **d**, and reverse, **e**, current from 100 locations at the surface. Current spikes at the positive switching voltage were detected in almost every ferroelectric cycle. The curves were measured in ultrahigh vacuum at 3×10^{-10} Torr using conductive atomic force microscopy (c-AFM).

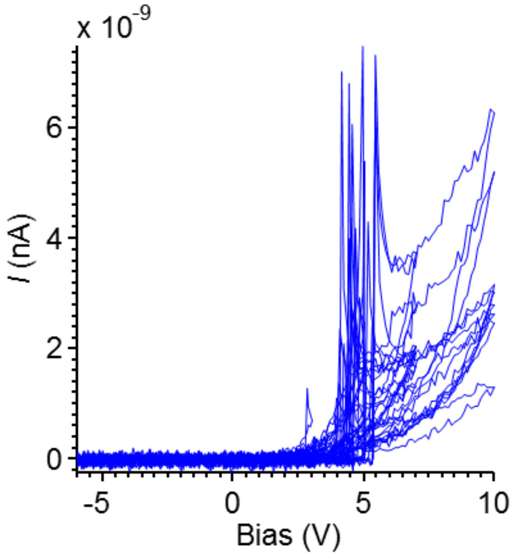

Supplementary Figure 7 | Local dc current-*vs.*-voltage curves of annealed PZT film.

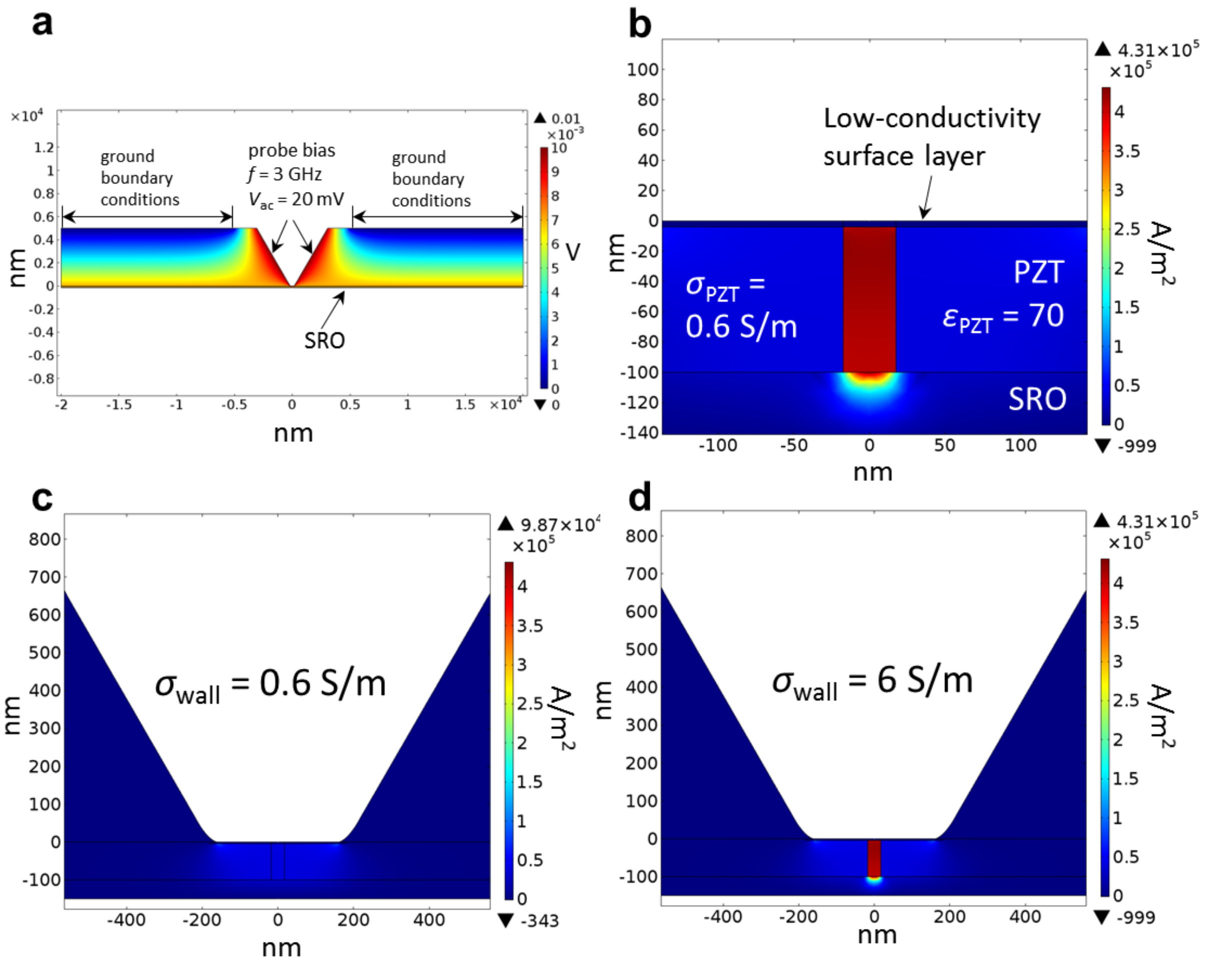

Supplementary Figure 8 | Modeling ac current distribution. (**a**) Layout of the finite-elements model with a map of calculated potential distribution in the PZT/SRO structure. SRO stands for $SrRuO_3$. (**b**) Close-up map of the z-component of the induced current density in the domain wall and adjacent portions of the PZT and SRO films. (**c**,**d**) Larger-scale maps of the *z*-component of the induced current density in the uniform domain bulk and in the presence of a domain wall, respectively. The material parameter values corresponding to the displayed maps are indicated in the figure panels.

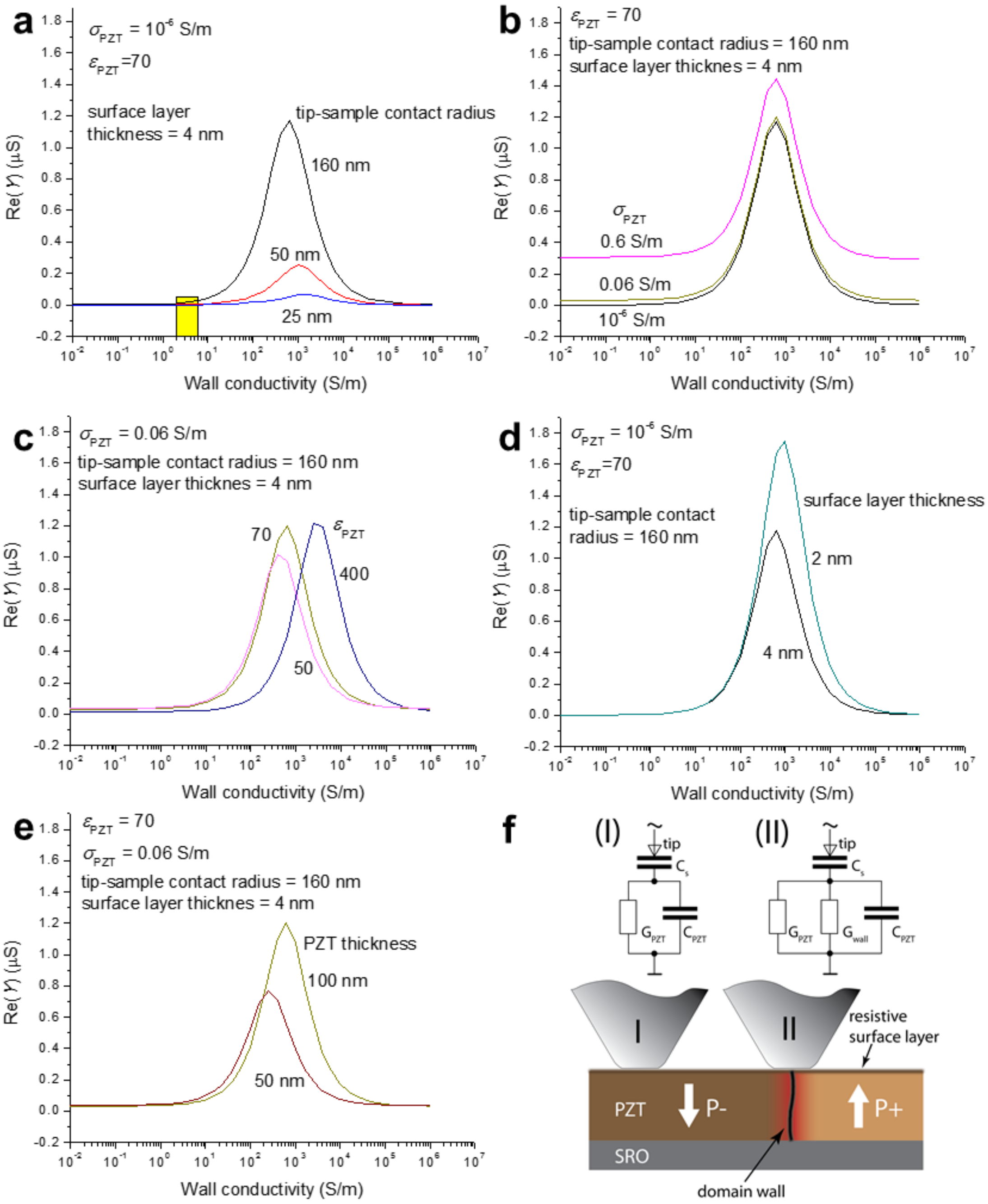

Supplementary Figure 9 | Numerical simulations of the tip-sample impedance. The figure shows only the real part of the impedance as a function of the domain wall conductivity for different material parameter values. The plots illustrate the effects of the parameters and show the range of the Re(Y) variability in response to the parameter change. (**a**) Parameter of the curve family is tip-sample contact radius. The change of the wall cross-section area on the surface in contact with the probe has been accounted in the calculations. The yellow rectangle denotes the range of the domain wall conductivity identified as closely matching the experimentally measured values. (**b-d**) Parameter of the curve families are conductivity of the

PZT film, permittivity of the PZT film, and thickness of the insulating surface layer, respectively. A larger set of similar curves was used to determine the values of ac conductivity from the measured values of the sMIM-*G* response. (**e**) A result of calculation suggesting a possible route for the optimization of the technique sensitivity *via* reduction of the PZT film thickness. (**f**) The diagrams on top show lumped-element high-frequency equivalent circuits corresponding to positions I and II of the probe over the PZT film. C_s is the capacitance of the resistive surface layer, C_{PZT} and G_{PZT} are the capacitance and conductance of the ferroelectric film, respectively, and G_{wall} is the conductance of the domain wall. Overall, the curves in **a-e** follow the behavior of the Re(*Y*) of the equivalent circuits shown in **f**.

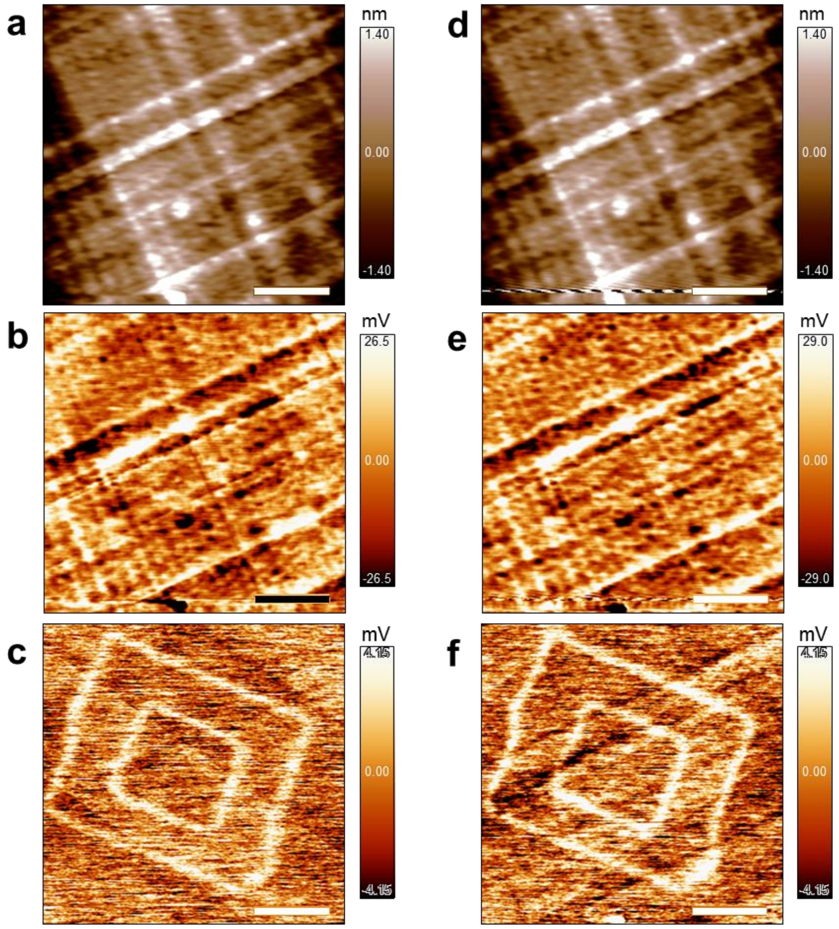

Supplementary Figure 10 | Sensitivity of the image contrast to the detection phase settings of the sMIM system. (**a**,**d**) Images of surface topography, (**b**,**e**) uncalibrated sMIM-*C* images, and (**c**,**f**) uncalibrated sMIM-*G* images of the same box-in-box domain structure. Images in **a-c** were acquired with the detector phase adjusted using an MC2 SMM calibration kit so that the signal in the sMIM-*G* channel from the 1 μm-diameter capacitors of the kit was zeroed. To acquire images in **d-f**, the phase setting was

changed by 3°. Note that the contrast in the sMIM-*C* images is mainly due to topographic cross-talk. The roughness of the film surface results in the varying tip-sample contact shape and consequently, varying tip-sample capacitance. With a correct setting of the detector phase, this contrast does not appear in the sMIM-*G* images as in **c**. The change of the phase setting leads to appearance of the capacitive signal in the sMIM-*G* image in **f**. However, still there is no visible signal from the domain walls in **e**. Both the sMIM-*C* images show a light contrast from domains. Apparently, a possible change of the material polarizability at the domain walls is too small to produce a strong enough sMIM-*C* signal change. In turn, the domain wall contrast in the sMIM-*G* channel is robust against inaccuracies of the detection phase setting (compare images **c** and **f** where the phase was set accurately and was 3° off, respectively). Scale bars are 2 μm.
